# *Announcement: *Global Road Safety Week, May 8–14, 2017

**DOI:** 10.15585/mmwr.mm6617a3

**Published:** 2017-05-05

Road traffic crashes are the world's leading cause of death among persons aged 15–29 years and the leading cause of death among U.S. teens aged 16–19 years (*1*). In the United States, 35,092 persons died in crashes during 2015; speeding was a factor in more than a quarter (27%) of these deaths (*2*). In October 2016, the National Safety Council, in partnership with the National Highway Traffic Safety Administration, the Federal Highway Administration, and the Federal Motor Carrier Safety Administration, announced the Road to Zero initiative, with the goal of eliminating traffic fatalities within 30 years (*3*).

As part of the Decade of Action for Road Safety 2011–2020, the Fourth United Nations (U.N.) Global Road Safety Week is May 8–14, 2017. This year’s theme is “SaveLives #SlowDown” with a focus on speed management and preventing speed-related injuries and deaths (*4**,**5*).

CDC supported the World Health Organization in preparing “Save LIVES: A road safety technical package,” which describes evidence-based measures that are most likely to impact road traffic deaths, including 22 interventions related to speed management, infrastructure design, vehicle safety, laws and their enforcement, emergency post-crash care and leadership on road safety (*6*).

In April 2016, the U.N. General Assembly adopted a resolution on “Improving global road safety” reaffirming the 2030 Agenda for Sustainable Development target numbers 3.6 (reducing global road traffic deaths and injuries by 50% by 2020) and 11.2 (providing access to safe, affordable, accessible and sustainable transport systems for all by 2030). The resolution acknowledges these targets and calls for action to reduce road traffic deaths and injuries as a pressing development priority (*7*).

Additional information about #SlowDown is available at http://www.who.int/violence_injury_prevention/publications/road_traffic/SlowDown_Days/en/. Additional information about U.N. Global Road Safety Week is available at https://www.unroadsafetyweek.org/en/home. Additional information about motor vehicle safety is available at https://www.cdc.gov/motorvehiclesafety/.
